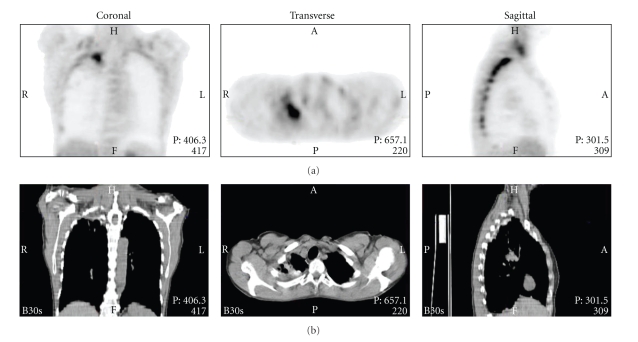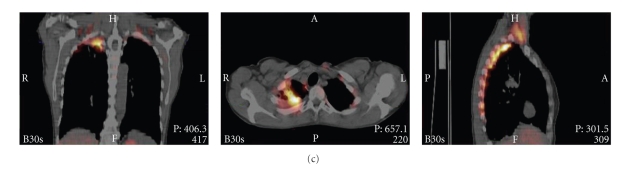# Pleural FDG Uptake More Than a Decade after Talc Pleurodesis

**DOI:** 10.1155/2009/650864

**Published:** 2009-07-30

**Authors:** Hilke Peek, Wouter van der Bruggen, Gijs Limonard

**Affiliations:** ^1^Department of Pulmonary Diseases, Canisius-Wilhelmina Hospital, 6525 SZ Nijmegen, The Netherlands; ^2^Department of Nuclear Medicine, Radboud University Nijmegen Medical Centre, 6525 GA Nijmegen, The Netherlands

## Abstract

Talc pleurodesis induces a strong local inflammatory reaction which can be detected by PET scan for years after the procedure. When patients undergo PET scanning in the workup of a suspected malignancy later in life, pleural FDG uptake may unnecessarily lead to an additional invasive diagnostic workup. We present two cases of positive pleural PET findings more than 10 years after talc pleurodesis, where we adopted a watchful waiting approach. Positive pleural PET findings as a result of prior talc pleurodesis should always be included in the differential diagnosis of pleural abnormalities.

## 1. Introduction

Talc pleurodesis is considered to be a safe and effective treatment modality in the management of (recurrent) spontaneous pneumothorax. In literature, increased pleural FDG-uptake on PET scanning is described for up to five years after the initial procedure [1–3]. Often, a malignant pleural disease is suspected, and patients undergo an extensive and invasive diagnostic workup. We present the cases of two patients whose PET scans revealed increased pleural uptake more than ten years after talc pleurodesis, without any signs of malignancy or infection at follow-up. 

## 2. Case Presentation

### 2.1. Patient 1

**Figure 1:**
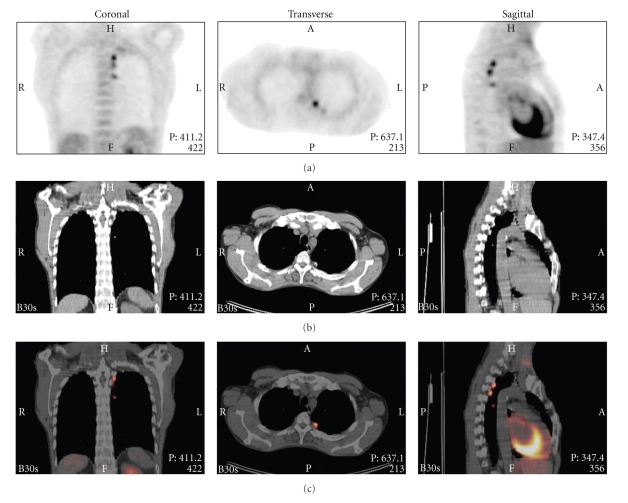


A 50-year-old man, heavy smoker, was found to have an abnormal chest X-ray, performed by his gastro-enterologist in the work up of abdominal complaints. His medical history was significant for a left-sided pneumothorax 10 years ago. At that time an uncomplicated thoracoscopic talc pleurodesis procedure was performed. Computed tomographic (CT) scan showed multiple nodular thickening of the posteromedial pleura on the left side. Positron emission tomography (PET) demonstrated high glucose uptake in all these lesions (Figure 1). Benign granulomatous inflammation after talc pleurodesis was suspected and we decided to perform follow-up CT-scans every 4 months. The areas of pleural FDG uptake have remained stable since and the patient is well, without any signs of malignancy or infection, 16 months after the initial PET scan. His presenting abdominal complaints were found to be the result of pancreatitis.

### 2.2. Patient 2

A 57-year-old woman presented with a lesion in the apex of the right upper lobe, found on routine chest X-ray. Medical history was significant for a right-sided pneumothorax for which she underwent talc pleurodesis 11 years ago, and a smoking status of more than twenty pack years. CT-scan showed signs of emphysema and multiple right-sided (sub)pleurally localised lesions, and a larger apical pleural plaque. All lesions had increased pleural FDG uptake (Figure 2). The abnormalities on CT and PET scan were deemed the result of the prior talc pleurodesis. Follow-up CT scans showed no changes of the pleural lesions, and the patient remains well 16 months after initial CT-scan.

## 3. Discussion

Talc pleurodesis is used widely for treatment of patients with persistent pleural effusions or pneumothorax not amenable to other treatment options. The pleural inflammatory response to talc administration is considered to be responsible for its ability to cause pleurodesis. Pathologic studies have indeed demonstrated a strong inflammatory reaction of both the visceral and parietal pleura following pleural talc administration, leading to the formation of pleural talc granulomata and eventually pleural fibrosis [1, 4].

Increased pleural FDG uptake on PET scanning after talc administration was first described by Murray et al. [1], ten months after pleurodesis. A more recent study showed the presence of PET-positive subpleural talc granulomas up to five years after pleurodesis [2]. The question indeed rises; how long will talc induced pleurodesis be detectable by PET? As these two cases suggest, this could be well for more than ten years.

In conclusion, these cases stress the importance of long-term persistent pleural inflammation after talc pleurodesis. As such, benign positive pleural PET findings in a patient with a history of talc pleurodesis should always be included in the differential diagnosis of pleural abnormalities, even if the original procedure was performed more than a decade ago.

## Figures and Tables

**Figure 2:**